# EUPopLink Country Report – Italy

**DOI:** 10.12688/openreseurope.23499.2

**Published:** 2026-05-29

**Authors:** Marta Lorimer

**Affiliations:** 1 Cardiff University School of Law and Politics, Cardiff, Wales, CF10 3AX, UK

**Keywords:** Italy; Populism; Euroscepticism; European Union, Europe

## Abstract

Italy has often been described as a ‘laboratory of populism’, however, its Eurosceptic turn is more recent. This country report, prepared as part of the EUPopLink COST Action, traces the relationship between populism and Euroscepticism in the Italian party system. It introduces the main populist and Eurosceptic actors in Italy, and considers the nexus between populism and Euroscepticism in the Italian context. It also considers mainstream responses to the rise of populist euroscepticism.

## 1. Introduction

Italy has often been described as a ‘laboratory of populism’, however, its Eurosceptic turn is more recent. Until the mid-1990s, both public and party-based Euroscepticism remained marginal phenomena ( Isernia, 2008; Roux & Verzichelli, 2010). At the party level, Euroscepticism was virtually absent until the mid-90s. From 1994 onwards, moderate Euroscepticism was expressed by Silvio Berlusconi’s Forza Italia and the post-fascist Alleanza Nazionale. Around the same time, the (at the time) ethno-regionalist Lega Nord also adopted an anti-EU position. Since then, Eurosceptic positions have hardened and become more diffuse, particularly amongst populist actors. At the level of electorates, Italian citizens shifted from being amongst the most enthusiastic supporters of the EU to some of its staunchest critics. Whereas in the early 1990s, support for the EU was overwhelmingly high (Eurobarometer data put it at around 80% - see Conti et al., 2020), it steadily declined only to drop strongly at the end of the 2000s. By 2011, only 41% of Italians thought that membership of the EU was a good thing ( Conti et al., 2020).

A key trigger for Italy’s Eurosceptic turn was the Eurozone crisis and its management, which brought the EU front and centre in public debate. The successive crises have contributed to keeping the EU a salient issue. Italy was at the forefront of responding to the so-called migration crisis and was hard-hit by the Covid-19 pandemic. As one of the EU’s largest member states, it also plays an important (if secondary compared to some of its EU counterparts) role in supporting Ukraine in its war against Russia. Although support for the EU has recovered some terrain since the Eurocrisis, it remains far from the heights of the early 1990s, and party-based Euroscepticism is now well entrenched.

This country report traces the links between party-based populism and Euroscepticims in Italy. It was prepared as part of the EUPopLink COST Action ( Andreadis & Vasilopoulou, 2026) following the EUPopLink Theoretical Framework and Guidelines ( Lorimer et al., 2026; EUPopLink, n.d.).

## 2. Populist political actors

There are several populist party actors in Italy and most of them are also Eurosceptic. Going from right to left, the main populist actors are:

• **Lega (League, formerly Lega Nord, populist radical right).** The Lega Nord started off as a union of autonomist movements representing the regions of the Italian north. Founded by Umberto Bossi in 1991, the party sought to obtain more autonomous decision-making powers for ‘the people’ in the northern Italian regions, against a ‘thieving’ Rome-centric ‘elite’. In 2013, the Lega elected a new leader, Matteo Salvini. Salvini has shifted the ideological profile of the party in nationalist, populist radical right direction ( Albertazzi et al., 2018). Its populist framework now centres opposition to EU elites rather than Italian elites. The Lega has been in government several times, and is part of Italy’s governing coalition since 2022. It has developed strong links with populist radical right parties in the EU and sits in the sovereigntist Patriots for Europe group in the European Parliament.
• **Fratelli d’Italia (Brothers of Italy, FdI, populist radical right).** Officially founded in 2012 from a splinter of the right-wing coalition People of Freedom, FdI is a ‘rooted newcomer’ ( Baldini et al., 2023): it is the successor of the conservative Alleanza Nazionale, itself the successor of the neofascist Movimento Sociale Italiano. Following a few years of political irrelevance, the party experienced a surge in popularity since 2019 due to the weakening of the Lega’s support and its role as sole official opposition party to Mario Draghi’s government. Giorgia Meloni, the party leader, is currently the head of a coalition government with the Lega and Forza Italia. FdI combines a broadly liberal economic agenda with nativist positions on immigration and a propensity for law-and-order policies. It is a leading member of the European Conservatives and Reformist group in the European Parliament.
• **Forza Italia (Go Italy, borderline case).** Forza Italia was founded in 1993 by late media tycoon Silvio Berlusconi and can be classified, at least under his leadership, as an economically liberal, right-wing populist party ( Bobba & McDonnell, 2016; Mudde, 2007; Verbeek & Zaslove, 2016). It fits none of the three categories (left-wing populist, populist radical right, valence populist) used in this study. The qualifier of populism mainly speaks to Berlusconi’s style of politics. A former entrepreneur, he positioned himself as an outsider and displayed an ‘anti-political’ stance. Forza Italia was also extremely leader-centric, and promoted full frontal attacks against liberal-democratic institutions ( Albertazzi & Mueller, 2013). Forza Italia has led government several times and until the early 2010s, it was the dominant force in the Italian right. Since Berlusconi’s retreat from politics, it has lost its hold on the Italian centre-right and moderated its populism. Forza Italia is a member of the European People’s Party and is well-integrated into the EU’s structures.
• **Movimento Cinque Stelle (Five Star Movement, M5S, valence populist party, sometimes left-wing populist).** It was founded in 2009 by comedian Beppe Grillo but became electorally successful in the years between 2013 and 2018. In 2018, it succeeded in forming a government first with the Lega and then with the centre-left Partito Democratico (Democratic Party, PD). Since 2018, it has experienced a steep decline in popularity although it is still electorally successful in Southern Italy ( Garzia, 2023). Its main agenda centres on a mix of anti-establishment claims and broadly progressive socio-economic policies. The M5S has been less successful than its populist radical right counterparts in establishing transnational collaborations with likeminded parties and currently sits with The Left Group.

## 3. Positions on ‘Europe’ and the Populism-Euroscepticism nexus

The Lega, FdI and the M5S are the main populist Eurosceptic actors in the Italian context. Although Forza Italia has expressed Eurosceptic positions in the past, it has shifted into progressively more pro-EU territory ( Biancalana et al., 2024), leading to its exclusion from the current analysis. Other smaller party actors and groups express consistently populist and sovereigntist Eurosceptic positions (e.g., parties such as Italexit or Democrazia Sovrana Popolare), however, they have remained electorally unsuccessful. The centre and the Left, for their part, remain mostly pro-European.

The Lega is the most consistently Eurosceptic of the three populist Eurosceptic parties. Although not opposed to the principle of European collaboration, it is both opposed to the current practice of European integration and to extending it to new areas. It opposes both polity and policy aspects of the European Union. In polity terms, in the 2024 EU elections, it ran with the slogan ‘More Italy, less Europe’, a position that well-reflects its scepticism not just of future integration, but also of the current structures of the EU. This position is consistent with those it defended in previous years. In terms of policies, like the M5S, it toyed with the idea of having a referendum on the Euro (although it abandoned this commitment) and generally expressed strong criticism against EU governance in the areas of economics and migration. In line with its radical right profile, the Lega has strongly opposed the Schengen area and advocated for breaking EU rules on migration. It has also developed a populist critique of EU institutions (especially the Commission) as overly bureaucratic, detached from citizens and against ‘common sense’. Its posters for the 2024 EU election are instructive of the kind of policy aspects the party picks up: they include the ‘more Italy, less Europe’ mentioned above, but also a commitment to defend made in Italy by ‘stopping artificial meat and insects’, and stopping ‘green Euro-follies’, ‘Islamic extremism’ and ‘sending Italian soldiers to fight in Ukraine’. Overall, the Lega’s critique of the EU is in line with its populist radical right profile. As could be expected by a nativist party, it views the EU as a threat to the identity, sovereignty and economic well-being of the Italian nation. At the same time, it also articulates its opposition in populist terms, identifying EU elites as enemies of the Italian people.

If the Lega has been quite consistent in its positions, FdI has shifted its views since it was founded. Although it remains Eurosceptic, it has moved from stark opposition to the EU to a more constructive approach. In the first EU election in which it took part, FdI advocated for a dissolution of the Eurozone or for Italy to leave the Eurozone, as well as for suspending Italy’s participation in EU economic policies such as the Fiscal Compact, European Stability Mechanism, and Stability and Growth Pact. It also critiqued European bureaucracy and ‘Eurowaste’, while demanding more collaboration in matters of migration. In line with its populist radical right profile, it also advocated for the protection of Europe’s Christian roots and the value of life, individuals and the family. The 2019 euromanifesto amped up the polity critique, opening on the need to reform the EU in the direction of a European confederation of sovereign states. The manifesto opposed the EU in populist and nationalist terms, critiquing Eurocrats and technocrats, while centring the need to protect Italy’s national interests. It also included elements of policy critique, particularly in its opposition to austerity and the Euro. Many of the policies, however, have more to do with domestic priorities and do not seem to have an EU angle to how to implement them. Since taking over government responsibilities in 2022, FdI has moderated its opposition. It adopted a ‘Euro-realist’ approach of engaging with the EU institutions in various policy areas, while remaining critical of certain polity-aspects and particularly of the EU’s effects on national sovereignty. It also, however, insists more on the notion of changing the institutions from within and shifting the EU towards its own priorities. Its approach, as presented in the manifesto, is to demand that the EU ‘do less, but better’. In this sense, while it expresses some polity-scepticism, particularly when advocating for a confederal form of European integration that will ‘respect the principles of proportionality and subsidiarity’ ( Fratelli d’Italia, 2024), it does not oppose EU collaboration per se and considers the EU should focus on the ‘great questions of our times’ (such as foreign policy). Its programme reflects its broad populist radical right priorities (including a focus on food sovereignty, pro-natality policies, opposition to green policies and migration), but with some reference to liberal economic policies and individual freedoms. In general, the nativist frame dominates over the populist one in the expression of Euroscepticism. EU institutions are occasionally criticised for being bureaucratic and unelected, but the frame of ‘people’ versus ‘elites’ is not explicitly used. Rather, the focus is on Italy’s identity and national interest in the EU construction.

The Movimento Cinque Stelle, like FdI, has also shifted positions over the years. The M5S has never fully opposed the principle of EU integration, although it has often expressed scepticism about the practice and, to a lesser extent, future of the EU. In its early years, it adopted both policy and polity Euroscepticism and was amongst the staunchest critics of EU integration, particularly in virtue of a strong ‘no Euro’ current within the party. In its 2014 EU election manifesto, although it did not advocate leaving the EU, it opposed austerity policies, proposed a referendum on the Euro and suggested a reform of the EU’s architecture. By 2019, its positions had mellowed. Although it still opposed EU-imposed austerity measures, it no longer advocated for Italy to leave the Euro and broadly accepted Italy’s membership of the EU. It also sought to ‘upload’ some of its domestic policies to the EU level – e.g., by advocating for an EU minimum wage. By the 2024 elections, while it remained critical of several EU policies (e.g., austerity measures) and to some extent of the EU’s current institutional architecture (suggesting, amongst other things, to reform the treaties in a more democratic direction), its critiques were broadly inserted into a framework of reforming the EU and pursuing more ambitious integration in certain areas (such as socio-economic rights, green policies or European identity). Massetti (2023) defines the M5S’s profile as a ‘critically Europhile’ one. When the EU is criticised, it is often done in populist terms. The European ‘establishment’ is attacked for being in cahoots with big financial interests, at the expense of European citizens. Parties in Europe are accused of colluding with one another to pursue their interests instead of doing what is good for citizens. In line with its early ‘valence populist’ agenda, the M5S also includes references to some of its core anti-corruption values as well as to direct democracy in its European programmes. These populist frames, it should be noted, have been toned down in recent years, and the 2024 EU election programme is significantly less populist in content than previous programmes.

As noted above, many of Italy’s populist Eurosceptic parties have changed position over time. It is hard to ascribe this to a response to any one of the EU’s most recent crises, however, crises did have an impact on some of these changes ( Braun et al., 2019). Brexit did not generate much debate, and most of them had, at most, advocated for leaving the Eurozone rather than the EU so it did not become an inspiration (or cautionary tale, for that matter). The Covid crisis did generate some movement ( Capati et al., 2024), especially for the M5S. Its shift to more pro-EU positions can be understood both as the result of taking on governing responsibilities, and as a response to the EU’s shift in priorities following the pandemic. The arrival of large amounts of EU funding to Italy countered the party’s narrative of the EU as an agent of austerity and prompted them to shift to a line more supportive of a ‘social’ Europe. The war in Ukraine was fitted into the parties’ agendas, but in different directions (see Capati & Trastulli, 2025 for a more extensive discussion). The M5S primarily started defending ‘peace’ and opposing further EU action. FdI took a pro-Ukraine stance and participated actively in EU efforts in the area. The Lega adopted a more ambivalent stance. It has declared its support for Ukraine, but also tried to limit European and particularly Italian involvement in the conflict and sought to undermine governmental action in this area. Finally, Trump’s election has generated enthusiasm in the Lega, but put FdI in a difficult position. While Giorgia Meloni initially had good relations with Trump, these have soured over the second year of his presidency.

## 4. Responses to Euroscepticism and consequences

When it comes to the political mainstream, Italy is a rather unique case in that its political spectrum is dominated by populist actors. It is therefore hard to identify a clear ‘mainstream’ that populist Eurosceptics might be opposing, and much of the competition over Europe has happened within the populist party family. The main, non-populist mainstream party is the centre-left PD. The PD is broadly pro-EU and has generally taken an adversarial stance to populist Euroscepticism ( Fifi, 2025). Forza Italia has been generally quite ambivalent towards European integration, adopting both accommodative and adversarial stances. In recent years, the latter have dominated, with Forza Italia adopting an increasingly pro-EU profile. This can be seen as part of an attempt to craft a more distinctive position vis-à-vis its right-wing competitors. Italy also has a wide array of smaller centrist and broadly pro-EU parties (e.g., Italia Viva, Azione, Europa+) whose positions could be classed as adversarial.

In terms of the concrete implications of the growth of party-based Euroscepticism, they remain quite limited. The EU question has certainly been politicised in Italy. Several parties have expressed diverging positions on the EU, used it political competition ( Capati, 2025), and Italy’s public opinion is now more Eurosceptic than in the past. However, this has not had significant political repercussions. For much of the period since the Eurozone crisis began, Italy was governed by Europhile governments and when in government, even the EU’s staunchest critics have failed to produce concrete changes in policy direction. This was blatantly visible when the M5S – Lega coalition government sought to negotiate the conditions of Italy’s 2019 budget with the Commission, and failed to obtain any concessions. These difficulties in reforming the EU are partially due to the structural issues faced by Italian parties wishing to push EU policy in new directions. Reforming the EU is notoriously difficult, and even more so for a Eurozone country with a weak economy. Italy needs continued support from the European Union to maintain trust from investors, meaning it has little leeway in negotiating conditions such as those demanded by populist Eurosceptic actors. Italy has also, over the last decade of crises, often demanded solidarity from its EU neighbours, be it in the context of the migration crisis or the Covid-19 crisis. Overall, although Italian populist Eurosceptic leaders have in the past been able to slow down or challenge EU processes (see e.g., Zaun and Ripoll Servent, 2023), they have had a marginal impact overall.

Perhaps more interestingly, Italian populist Eurosceptic parities have been best able to affect EU politics when they moderated their stances in their dealings with the EU. Giorgia Meloni is a case in point. By adopting a constructive attitude towards Ukraine and developing links with the European Commission’s president Ursula von der Leyen, she has been able to shape some of the EU’s thinking around migration. Although her influence on the institutions is often overstated (as she herself discovered when seeking to gain influence for ECR in the von der Leyen Commission), she is certainly less ostracised than her populist predecessors.

## 5. Short conclusion

The landscape of populism and Euroscepticism in Italy is rich. As the report has shown, populist Euroscepticism is widespread in Italy, but it presents itself in various iterations. On the right, two different populist radical right parties present Eurosceptic positions, but they vary in the degree of opposition to the EU they display. The M5S, for its part, presents a clear populist profile, but has shifted in terms of its position towards the European Union. Even though there is variation in the degrees of euroscepticism expressed by populist parties in Italy, they all broadly accept the principle of EU integration while critiquing its current practice and its future ( Vasilopoulou, 2018). They also all adopt both ‘polity’ and ‘policy’ scepticism ( Mair, 2007), but their opposition can be classed primarily as ‘soft’ rather than ‘hard’ ( Szczerbiak & Taggart, 2008). Importantly, the analysis above has shown that Euroscepticism is far from being a static feature of political parties, indicating the need for further research to explain how and why populist parties change their views.

Overall, however, while populist Euroscepticism is well-represented in Italy, it has not had a strong effect in terms of Italy’s policies towards the EU, partially due to the dominance of Europhiles for much of the last decade and because of the moderating effect that government has had on some populist actors. This suggests that although Euroscepticism is an ideological feature that populist parties embrace, they do not necessarily follow through with their anti-EU agenda, or even stick to it over the long term. There may, in this sense, be a gap between Eurosceptic rhetoric and Eurosceptic practice that is worth exploring further.

## Ethics and consent

Ethical approval and consent were not required.

## Data Availability

No data are associated with this article.
