# Supplementary material for: EUPopLink Country Report – Italy
Source: Open Res Eur. 2026 May 29;6:131. Originally published 2026 Apr 21. [Version 2] doi: 10.12688/openreseurope.23499.2 (PMC13168876; doi:10.12688/openreseurope.23499.2)
Supplement: Peer review history

# Reviewer report: Reviewer response for version 2

**DOI:** 10.21956/openreseurope.26046.r75167

**Published:** 2026-06-04

**Authors:** Nika Gigauri

**Affiliations:** 1 GIPA – Georgian Institute of Public Affairs, Tbilisi, Georgia

Thank you for addressing the recommendations. The updated version effectively incorporates the theoretical framework, and I am happy to endorse this final draft.

Is the case presented with sufficient detail to be useful for teaching or other practitioners?

Yes

Is the work clearly and accurately presented and does it cite the current literature?

Yes

If applicable, is the statistical analysis and its interpretation appropriate?

Not applicable

Are all the source data underlying the results available to ensure full reproducibility?

No source data required

Are the conclusions drawn adequately supported by the results?

Yes

Is the background of the case’s history and progression described in sufficient detail?

Yes

Reviewer Expertise:

Political Science, Party Politics, Populism, and Euroscepticism.

I confirm that I have read this submission and believe that I have an appropriate level of expertise to confirm that it is of an acceptable scientific standard.

# Reviewer report: Reviewer response for version 2

**DOI:** 10.21956/openreseurope.26046.r75168

**Published:** 2026-06-04

**Authors:** Andrea Capati

**Affiliations:** 1 Luiss University, Rome, Italy

All comments have been satisfactorily addressed and the article is ready for indexing.

Is the case presented with sufficient detail to be useful for teaching or other practitioners?

Yes

Is the work clearly and accurately presented and does it cite the current literature?

Partly

If applicable, is the statistical analysis and its interpretation appropriate?

Not applicable

Are all the source data underlying the results available to ensure full reproducibility?

Partly

Are the conclusions drawn adequately supported by the results?

Yes

Is the background of the case’s history and progression described in sufficient detail?

Yes

Reviewer Expertise:

European integration, party competition, EU economic governance, EU financial assistance, EU crises

I confirm that I have read this submission and believe that I have an appropriate level of expertise to confirm that it is of an acceptable scientific standard.

# Reviewer report: Reviewer response for version 1

**DOI:** 10.21956/openreseurope.25438.r72905

**Published:** 2026-05-29

**Authors:** Antonella Seddone

**Affiliations:** 1 University of Turin, Turin, Italy

This country report offers a well-structured account of the relationship between populism and Euroscepticism in the Italian party system. The reconstruction of party trajectories across multiple electoral cycles is careful and nuanced, and the report succeeds in capturing the considerable variation among populist actors, both in the degree of their opposition to the EU and in the direction of their evolution over time.

 Three observations may help strengthen the contribution.

 The report would benefit from a brief engagement with the literature on the shift from permissive consensus to constrained dissensus in European integration. This framing matters for the Italian case because it helps explain why Euroscepticism became electorally viable when it did, not as a linear outgrowth of pre-existing ideological commitments, but as a consequence of the politicisation of the EU issue in a context where public opinion had previously been largely indifferent. The Eurozone crisis accelerated a process of EU politicisation already underway, converting a gradual erosion of diffuse public support into an explicit axis of party competition. The literature on the shift from permissive consensus to constrained dissensus captures this dynamic well, framing politicisation not as a sudden rupture but as the cumulative outcome of rising EU visibility and contestation over a longer arc.

 A related gap concerns the domestic political environment that made anti-elite rhetoric so resonant in Italy before the EU became a salient target. The report traces the emergence of Eurosceptic positions among populist actors but does not fully account for the cultural and historical substrate in which those positions took root. The collapse of the First Republic following the Tangentopoli investigations of the early 1990s left a lasting imprint on Italian political culture, consolidating a widespread disposition toward anti-establishment politics that predated, and in important ways facilitated, the subsequent uptake of Eurosceptic frames. The trajectory of the Lega is instructive in this regard. Its original anti-elite mobilisation targeted the Italian state, Rome, and what its rhetoric cast as the southern-subsidised political class. The pivot toward the EU as the primary antagonist came later, at the point when that domestic target had been substantially exhausted as a resource for political differentiation. Reading the Lega's Euroscepticism as a relocation of the anti-elite frame, rather than an independent ideological commitment, has implications for how we interpret the stability and coherence of such positions over time, and would add analytical depth to what is already a perceptive account of the party's evolution.

 A third observation follows directly from the preceding one and concerns the mechanisms behind party-level moderation. The report documents with care the softening of Eurosceptic positions in the cases of FdI and M5S following their entry into government, but stops short of providing an explicit account of why this occurs. The structural logic behind the empirical pattern deserves to be spelled out, because it bears directly on the broader question of whether Euroscepticism functions as ideological conviction or as political strategy. Challenger parties operate under a fundamentally different opportunity structure than governing parties. Eurosceptic claims carry low cost and high electoral salience when made from opposition, but become structurally constrained once in office, given bond market pressures, dependence on EU transfers, and the reputational requirements of international credibility. FdI's shift toward what the report aptly calls a Euro-realist posture is, on this reading, less a moderation of underlying preferences than an adaptation to an altered set of incentives and constraints. Making this mechanism explicit would strengthen the report's contribution to the broader debate on populist parties in government and would also provide a more satisfying account of why Eurosceptic rhetoric and Eurosceptic practice so frequently diverge, the gap the report identifies in its conclusion but does not fully explain.

 These are suggestions for deepening an already solid contribution. The report succeeds in its primary aim of mapping the populism-Euroscepticism nexus in a complex and consequential case, and the empirical material is handled with appropriate care and discrimination.

Is the case presented with sufficient detail to be useful for teaching or other practitioners?

Yes

Is the work clearly and accurately presented and does it cite the current literature?

Yes

If applicable, is the statistical analysis and its interpretation appropriate?

Not applicable

Are all the source data underlying the results available to ensure full reproducibility?

No source data required

Are the conclusions drawn adequately supported by the results?

Yes

Is the background of the case’s history and progression described in sufficient detail?

Yes

Reviewer Expertise:

Political Science, Political parties, Public opinion, Political Communication

I confirm that I have read this submission and believe that I have an appropriate level of expertise to confirm that it is of an acceptable scientific standard.

# Reviewer report: Reviewer response for version 1

**DOI:** 10.21956/openreseurope.25438.r72907

**Published:** 2026-05-12

**Authors:** Francesco Alicino

**Affiliations:** 1 University LUM, Bari, Italy

The article offers a concise overview of the relationship between populism and Euroscepticism in contemporary Italy. It reconstructs the evolution of party-based Euroscepticism from the 1990s to the present and examines how different populist actors – namely Lega, Fratelli d'Italia (FdI), Forza Italia (FI), and Movimento Cinque Stelle (M5S) – have articulated distinct forms of opposition to European integration, thereby contributing to the rise of populist Euroscepticism.

 One of the principal strengths of the article lies in its ability to synthesize a broad and complex political trajectory within a relatively concise format. The historical contextualization is particularly effective: the author demonstrates how Italy evolved from one of the most Europhile Member States into a political environment in which Eurosceptic narratives became increasingly normalized following the Eurozone crisis. The discussion of successive crises – including migration, the Covid-19 pandemic, and the war in Ukraine – adds further analytical depth by situating Italian developments within broader European dynamics.

 The sections devoted to the main party actors are among the strongest parts of the article. The reconstruction of the ideological evolution of Lega, FdI, FI, and M5S is nuanced and balanced, avoiding simplistic categorizations. In particular, the analysis effectively captures the distinction between nationalist-populist Euroscepticism and more fluid, issue-based forms of Euroscepticism. The article also succeeds in illustrating how participation in government has moderated certain anti-EU positions, especially in the cases of FdI and M5S. This represents an important contribution to the broader scholarly debate concerning the strategic adaptability of populist actors once in government.

 At the same time, several aspects of the article could be further strengthened. First, although the article repeatedly refers to the distinction between polity and policy-based Euroscepticism, these conceptual categories could be defined more explicitly at the outset and applied more systematically throughout the analysis. Moreover, the article could engage more directly with the theoretical implications of the transformations described, particularly in relation to FdI and M5S, whose trajectories suggest that Euroscepticism is neither static nor ideologically uniform. The moderation of FdI following its entry into government and the gradual shift of M5S toward a more “critical Europhile” position deserve more explicit theorization in relation to debates on party institutionalization, strategic adaptation, and the constraints imposed by both national and supranational governance structures.

 A further aspect that remains somewhat underdeveloped concerns the level of public understanding of the European Union (EU) within the Italian context. While the article convincingly reconstructs party-based Eurosceptic narratives, it devotes comparatively less attention to the broader social and cognitive environment in which such narratives gain electoral resonance. In particular, the article could benefit from a more explicit discussion of the widespread uncertainty surrounding the actual functioning of EU institutions, the relationship between European law and the Italian constitutional order, and the concrete impact of EU decision-making on Italy’s economic, social, and financial governance. Issues concerning the primacy of EU law, direct effect, and the interaction between national and supranational competences remain largely absent from public debate, despite their central relevance for understanding both support for and opposition to European integration.

 This omission is analytically significant because the nexus between populism and Euroscepticism often appears to rely on highly simplified, if not distorted, representations of the European Union. In many cases, the EU becomes a flexible political construct onto which national actors project contradictory expectations and frustrations. Parties commonly labelled as populist frequently invoke the need for both “more Europe” and “less Europe” depending on the issue area and policy field involved, irrespective of the EU’s actual competences and institutional functions in those domains or, more broadly, of the role played by Member States in governing them. At the same time, these actors often oppose the institutional and sovereignty transfers that would be necessary to implement genuinely supranational solutions. In other words, they criticize the EU for allegedly undermining national identity and sovereignty, yet continue to appeal to European institutions whenever national governments prove incapable of addressing sensitive issues such as migration crises, economic instability, or geopolitical insecurity. In this sense, the EU frequently functions as a convenient scapegoat for domestic political failures, particularly in contexts where public knowledge of EU institutional mechanisms remains limited.

 The contemporary Italian debate offers several illustrations of this ambiguity. Recent statements by Prime Minister Giorgia Meloni advocating a “new political Europe” while simultaneously rejecting further transfers of sovereignty exemplify a broader contradiction that characterizes not only populist radical-right actors but also ostensibly pro-European forces. Calls for a stronger and more strategic Europe are often disconnected from the institutional reforms that such ambitions would necessarily require. This tension is particularly visible in areas such as foreign policy, defence, migration governance, technological regulation, and economic coordination, where Member States continue to resist deeper integration while simultaneously criticizing the EU's perceived inefficiency. The result is a recurrent oscillation between Europhile and sovereignist discourses, which helps explain both the ideological flexibility and strategic repositioning of parties such as Lega and Fratelli d’Italia and the ambiguities of formally pro-European actors such as M5S and the Partito Democratico (PD). Indeed, these parties and their leaders often appear to embody what might be described as an “identity of contradictions”: they are pro-European yet also moderately sovereignist; they reject nationalism while simultaneously defending the notion of a "Europe of nations"; they criticize the fragmentation of the EU in matters of foreign policy, security, and defence, yet rarely explain how a common European army, integrated police cooperation, or a unified foreign policy could realistically be established.

 These observations, however, do not detract from the overall quality of the article.  The article offers a valuable contribution both to specialists working on Italy and to scholars interested more broadly in comparative populism and European integration.

Is the case presented with sufficient detail to be useful for teaching or other practitioners?

Yes

Is the work clearly and accurately presented and does it cite the current literature?

Yes

If applicable, is the statistical analysis and its interpretation appropriate?

Not applicable

Are all the source data underlying the results available to ensure full reproducibility?

Partly

Are the conclusions drawn adequately supported by the results?

Yes

Is the background of the case’s history and progression described in sufficient detail?

Yes

Reviewer Expertise:

Constitutional Law and EU Law

I confirm that I have read this submission and believe that I have an appropriate level of expertise to confirm that it is of an acceptable scientific standard.

# Response

**Published:** 2026-05-18

**Authors:** Marta Lorimer

**Affiliations:** Cardiff University, Cardiff, Wales, UK

Thank you for your positive evaluation of the country report on Italy and for the detailed and constructive feedback provided. Below, I detail how I amended the report in response to the recommendations made.

**Clarifying the conceptual categories** *'First, although the article repeatedly refers to the distinction between polity and policy-based Euroscepticism, these conceptual categories could be defined more explicitly at the outset and applied more systematically throughout the analysis.'*

- **Response:** Thank you for this suggestion. The present report was prepared as part of the EUPopLink COST Action (Andreadis and Vasilopoulou, 2026) following the EUPopLink Theoretical Framework and guidelines (Lorimer et al., 2026). In the interest of keeping the reports concise and consistent, the full definition of all conceptual categories is provided in the guidelines (available here: https://www.eupoplink.eu/node/17 and in Lorimer et al. 2026) and not repeated in individual country reports, as this would take away space from the empirical account that is central to the country case studies. To clarify this further, I have added a link to the guidelines at the beginning of the report. I have also sought to link the findings back to the conceptual categories in the conclusion (a point raised by Reviewer 1 as well).

 
**Considering further the theoretical implications of the study** *'Moreover, the article could engage more directly with the theoretical implications of the transformations described, particularly in relation to FdI and M5S, whose trajectories suggest that Euroscepticism is neither static nor ideologically uniform. The moderation of FdI following its entry into government and the gradual shift of M5S toward a more “critical Europhile” position deserve more explicit theorization in relation to debates on party institutionalization, strategic adaptation, and the constraints imposed by both national and supranational governance structures.'*

- **Response: **While agreeing that discussing further the theoretical implications of the study would be valuable and interesting, this is beyond the scope of the country case studies. The country case studies are one part of the overall EUPopLink project. Their narrow objective is to provide an empirical account of the link between populism and Euroscepticism in European countries. Drawing more extensive theoretical implications, including along the lines suggested, will be the task of the next steps of the project. I have, however, added a sentence in the conclusion to stress the dynamic nature of Euroscepticism and the need for future research to expand upon and explain this finding.

 
**Party positions and public understanding of the EU**

*‘A further aspect that remains somewhat underdeveloped concerns the level of public understanding of the European Union (EU) within the Italian context. While the article convincingly reconstructs party-based Eurosceptic narratives, it devotes comparatively less attention to the broader social and cognitive environment in which such narratives gain electoral resonance. In particular, the article could benefit from a more explicit discussion of the widespread uncertainty surrounding the actual functioning of EU institutions, the relationship between European law and the Italian constitutional order, and the concrete impact of EU decision-making on Italy’s economic, social, and financial governance. Issues concerning the primacy of EU law, direct effect, and the interaction between national and supranational competences remain largely absent from public debate, despite their central relevance for understanding both support for and opposition to European integration. […]’*

- **Response: **This comment offers a thoughtful and valuable reflection on the link between party-based Euroscepticism and voter-based Euroscepticism, an aspect that this country report admittedly does not engage with. However, this is a comment that the country report itself cannot do justice to or fully engage with. The country reports are meant to centre on the supply-side of populism and Euroscepticism and offer a concise summary of this specific aspect of the populism/euroscepticism nexus. Other parts of the EUPopLink project look more closely at voters, and could, potentially, offer further points of reflection on this comment. To clarify the focus on the supply-side, I have nonetheless added a direct reference to it in the introduction.

# Reviewer report: Reviewer response for version 1

**DOI:** 10.21956/openreseurope.25438.r73327

**Published:** 2026-05-07

**Authors:** Andrea Capati

**Affiliations:** 1 Luiss University, Rome, Italy

The report addresses an interesting and relevant topic, focusing on the relationship between Italy’s populist parties and their Euroscepticism. The report is well structured and clear in its language. The empirical analysis is detailed and accurate. It provides a comprehensive overview of populist parties in Italy, their positions on the European Union (EU) and the implications for political competition with mainstream parties. To strengthen the report, I have three main recommendations.

 While Forza Italia has often been associated with a personalistic, leader-centred and media-driven form of party organisation, it is less clear whether it can be straightforwardly classified as a populist party in the same sense as the Movimento 5 Stelle or the Lega. In particular, the report should clarify whether Forza Italia has historically articulated a sufficiently strong anti-elitist discourse to justify its inclusion in the populist party family. If the author’s argument is that Forza Italia represents a specific type of populism, for instance centred on Berlusconi’s direct relationship with ‘the people’, this should be mentioned more explicitly and backed by empirical evidence. Alternatively, the report could rely more explicitly on the relevant academic literature to justify this categorisation. At present, the inclusion of Forza Italia risks appearing somewhat asserted rather than fully justified. 

 Second, while the report provides a convincing descriptive account of Italian populist parties’ evolving stances towards the EU, it somewhat neglects the drivers behind such an evolution. In particular, the report argues that ‘it is hard to ascribe this [changed position] to a response to any of the EU’s most recent crises’. However, there is a large literature demonstrating that the EU’s own response to these crises – most notably the Covid-19 pandemic and the Russian war on Ukraine – has indeed contributed to affecting populist parties’ positions on Europe (see Braun et al. 2019; Capati et al. 2024; and Capati and Trastulli 2025). The author mentions some of these findings in a late paragraph but these sources are never acknowledged. Incidentally, one of these works deals specifically with how mainstream and populist parties have used the EU issue during crises to attack each other (see Capati 2025), which is what the report refers to in its section 4. on responses to Euroscepticism and related consequences.

 Finally, the conclusion should be expanded. At this stage, the concluding section identifies some interesting patterns but does not sufficiently spell out the broader significance of the report’s contribution. The author could use the conclusion to clarify what the Italian case tells us about the relationship between populism and Euroscepticism more generally, and whether Euroscepticism should be understood as a stable ideological feature or as a more flexible political strategy. The conclusion could also reflect more explicitly on the implications of the findings for broader debates on party competition, crisis politics and the evolution of populist parties once they enter government or face EU-level constraints. The paper would also benefit from a more detailed indication of possible avenues for future research, including comparative work on other European countries or further analysis of the factors shaping populist parties’ changing positions on the EU.

Is the case presented with sufficient detail to be useful for teaching or other practitioners?

Yes

Is the work clearly and accurately presented and does it cite the current literature?

Partly

If applicable, is the statistical analysis and its interpretation appropriate?

Not applicable

Are all the source data underlying the results available to ensure full reproducibility?

Partly

Are the conclusions drawn adequately supported by the results?

Yes

Is the background of the case’s history and progression described in sufficient detail?

Yes

Reviewer Expertise:

European integration, party competition, EU economic governance, EU financial assistance, EU crises

I confirm that I have read this submission and believe that I have an appropriate level of expertise to confirm that it is of an acceptable scientific standard, however I have significant reservations, as outlined above.

## References

[1] : Responding to the crisis: Eurosceptic parties of the left and right and their changing position towards the European Union. *European Journal of Political Research* .2019;58(3) : 10.1111/1475-6765.12321 797-819 10.1111/1475-6765.12321

[2] : Italian party competition over the European Union’s financial response to COVID-19: a claims analysis. *Contemporary Italian Politics* .2025; 10.1080/23248823.2025.2488167 1-22 10.1080/23248823.2025.2488167

[3] : COVID-19 and party competition over the EU: Italy in Early Pandemic Times. *European Politics and Society* .2024;25(1) : 10.1080/23745118.2022.2095170 93-114 10.1080/23745118.2022.2095170

[4] : A quiet convergence: The 2022 full‐scale Russian invasion of Ukraine and Italian Parties' positions on the European Union. *Global Policy* .2025;16(1) : 10.1111/1758-5899.13466 28-44 10.1111/1758-5899.13466

# Response

**Published:** 2026-05-18

**Authors:** Marta Lorimer

**Affiliations:** Cardiff University, Cardiff, Wales, UK

Thank you for your detailed and constructive feedback. Below, I detail how I have addressed the three main recommendations made in the report. **Recommendation 1: Justifying the inclusion of Forza Italia**

*‘* *While Forza Italia has often been associated with a personalistic, leader-centred and media-driven form of party organisation, it is less clear whether it can be straightforwardly classified as a populist party in the same sense as the Movimento 5 Stelle or the Lega. In particular, the report should clarify whether Forza Italia has historically articulated a sufficiently strong anti-elitist discourse to justify its inclusion in the populist party family. If the author’s argument is that Forza Italia represents a specific type of populism, for instance centred on Berlusconi’s direct relationship with ‘the people’, this should be mentioned more explicitly and backed by empirical evidence. Alternatively, the report could rely more explicitly on the relevant academic literature to justify this categorisation. At present, the inclusion of Forza Italia risks appearing somewhat asserted rather than fully justified.’*

- **Response: **Thank you for raising this point. In response, I have added references to existing literature that classes Forza Italia under Berlusconi as a populist party (Bobba and McDonnell, 2016; Mudde, 2007; Verbeek and Zaslove, 2016). I have also added a clarification that the ‘populist’ aspect was most prevalent when Berlusconi was leader.

 
**Recommendation 2: Expanding the analytical insights with reference to recent literature**

 ‘Second, while the report provides a convincing descriptive account of Italian populist parties’ evolving stances towards the EU, it somewhat neglects the drivers behind such an evolution. In particular, the report argues that ‘it is hard to ascribe this [changed position] to a response to any of the EU’s most recent crises’. However, there is a large literature demonstrating that the EU’s own response to these crises – most notably the Covid-19 pandemic and the Russian war on Ukraine – has indeed contributed to affecting populist parties’ positions on Europe (see Braun et al. 2019; Capati et al. 2024; and Capati and Trastulli 2025). The author mentions some of these findings in a late paragraph but these sources are never acknowledged. Incidentally, one of these works deals specifically with how mainstream and populist parties have used the EU issue during crises to attack each other (see Capati 2025), which is what the report refers to in its section 4. on responses to Euroscepticism and related consequences.’

- **Response: **Thank you for this comment and for offering the opportunity to clarify the argument. I have revised this paragraph to state that while it is hard to ascribe party’s changed positions to any individual crisis, crises did likely have an impact on some of these changes. I have also integrated the suggested articles in the piece.

 
**Recommendation 3: Expanding the conclusion**

*‘* *Finally, the conclusion should be expanded. At this stage, the concluding section identifies some interesting patterns but does not sufficiently spell out the broader significance of the report’s contribution. The author could use the conclusion to clarify what the Italian case tells us about the relationship between populism and Euroscepticism more generally, and whether Euroscepticism should be understood as a stable ideological feature or as a more flexible political strategy. The conclusion could also reflect more explicitly on the implications of the findings for broader debates on party competition, crisis politics and the evolution of populist parties once they enter government or face EU-level constraints. The paper would also benefit from a more detailed indication of possible avenues for future research, including comparative work on other European countries or further analysis of the factors shaping populist parties’ changing positions on the EU.’*  

- **Response: **While agreeing that extending the conclusion would be potentially valuable and interesting, the length of the conclusion is constrained by the strict country report guidelines provided by the EUPopLink Working Group 1 (available here: https://www.eupoplink.eu/node/17). Country report authors were invited to keep the concluding section to a ‘brief summary and reflection on comparative idiosyncrasies of case and/or implications of analysis for broader research area’, as drawing more comprehensive (comparative) implications will be the goal of the project’s subsequent phases. In light of this consideration, and of the requirement to keep the report within the 3000 word limit set by the project, I have opted not to expand the conclusion extensively. I have, however, added a sentence to stress the importance of understanding Euroscepticism as a dynamic feature whose changing nature requires explaining. Further reflections on the implications of the findings of the reports, including along the lines suggested by the review, will be explored in future EUPopLink research.

# Reviewer report: Reviewer response for version 1

**DOI:** 10.21956/openreseurope.25438.r73333

**Published:** 2026-05-05

**Authors:** Nika Gigauri

**Affiliations:** 1 GIPA – Georgian Institute of Public Affairs, Tbilisi, Georgia

The article addresses a highly relevant and compelling topic, exploring the intricate relationship between populism and Euroscepticism within the Italian political landscape. The author provides a detailed and nuanced account of the similarities and differences among key political parties regarding their Eurosceptic stances. A particular strength of the work is its original presentation of the paradoxes and evolving trends that characterize Italian party politics, offering valuable insights into how these movements navigate European integration.

 The historical background and the progression of the party system are well-documented, and the article is clearly written. However, to further strengthen the paper and ensure it is fully scientifically sound, I have one primary recommendation:

 In the concluding section, the authors should provide a more explicit and systematic classification of the Italian parties within the established theoretical framework of Euroscepticism. While the descriptions are thorough, a clear, theory-driven categorization would help synthesize the findings and provide a more robust academic contribution. Specifically, linking the empirical observations more tightly to the theoretical types of Euroscepticism (e.g., hard vs. soft, or polity vs. policy-based) in the final discussion would clarify the overarching patterns identified in the study.

Is the case presented with sufficient detail to be useful for teaching or other practitioners?

Yes

Is the work clearly and accurately presented and does it cite the current literature?

Yes

If applicable, is the statistical analysis and its interpretation appropriate?

Not applicable

Are all the source data underlying the results available to ensure full reproducibility?

No source data required

Are the conclusions drawn adequately supported by the results?

Yes

Is the background of the case’s history and progression described in sufficient detail?

Yes

Reviewer Expertise:

Political Science, Party Politics, Populism, and Euroscepticism.

I confirm that I have read this submission and believe that I have an appropriate level of expertise to confirm that it is of an acceptable scientific standard.

# Response

**Published:** 2026-05-18

**Authors:** Marta Lorimer

**Affiliations:** Cardiff University, Cardiff, Wales, UK

Thank you for your positive evaluation of the country report on Italy and for your suggestion to improve the piece further. In response to the following recommendation concerning how the text could be improved:

*‘In the concluding section, the authors should provide a more explicit and systematic classification of the Italian parties within the established theoretical framework of Euroscepticism. While the descriptions are thorough, a clear, theory-driven categorization would help synthesize the findings and provide a more robust academic contribution. Specifically, linking the empirical observations more tightly to the theoretical types of Euroscepticism (e.g., hard vs. soft, or polity vs. policy-based) in the final discussion would clarify the overarching patterns identified in the study.’*

- **Response: **I have added a sentence in the conclusion that summarises the findings with respect to the theoretical categories discussed earlier in the text and in the theoretical framework for the project (Lorimer et al. 2026, EUPopLink n.d.).
